# Quality of heavy metal-contaminated soil before and after column flushing with washing agents derived from municipal sewage sludge

**DOI:** 10.1038/s41598-021-95441-5

**Published:** 2021-08-04

**Authors:** Barbara Klik, Zygmunt M. Gusiatin, Dorota Kulikowska

**Affiliations:** Department of Environmental Biotechnology, Faculty of Geoengineering, University of Warmia and Mazury in Olsztyn, 10-719 Olsztyn, Poland

**Keywords:** Biogeochemistry, Ecology, Environmental sciences

## Abstract

Removal of heavy metals (HMs) from soil is a priority in soil washing/soil flushing. However, for further management of remediated soil, it should be characterized in detail. This study presents, for the first time, an evaluation of soil quality after column flushing with new-generation washing agents (WAs) recovered from municipal sewage sludge (dissolved organic matter, DOM; soluble humic-like substances, HLS; soluble humic substances, SHS) and Na_2_EDTA as a standard benchmark. Sandy loam soil was spiked with industrial levels of Cu, Pb and Zn, then flushed in a column reactor at two WA flow rates (0.5 and 1.0 ml/min). Soil quality was assessed by determining both physico-chemical (pH, total HMs and their mobility, soil organic matter, OM, humic substances, HS and their fractions, macroelements) and biological indicators (dehydrogenase activity, DHA; germination rate, GR; and inhibition factors for roots and shoots of *Triticum aestivum*). Total residual HMs contents and HMs contents in the mobile fraction were significantly lower in soil flushed at 1.0 ml/min than in soil flushed at 0.5 ml/min. With all WAs, the decrease in Cu content was larger than that of the other HMs, however this HM most effectively was removed with DOM. In contrast, Pb most effectively was removed by HLS and Na_2_EDTA, and DOM should not be used to remediate Pb-contaminated soil, due to its very low effectiveness. Flow rate did not appear to affect the fertilizing properties of the soil, DHA activity or soil toxicity indicators. Soil flushing with all SS_WAs increased OM, HS, and exchangeable P, K and Na content in remediated soils, but decreased exchangeable Ca content, and in most cases, exchangeable Mg content. Soil flushing substantially improved DHA activity and GR, but only slightly improved the shoot and root inhibition factors.

## Introduction

Contamination of soils by heavy metals (HMs) is a common problem throughout the world, and the remediation technologies that enable permanent removal of HMs from soil can be ex-situ soil washing and in-situ soil flushing^1,2^. These technologies involve the application of washing agents (WAs) with various mechanisms of action, i.e., solutions of acids and salts, chelating agents or biosurfactants. One of the most commonly used WA, tested for three decades, is a synthetic chelator, i.e. EDTA^3^. In recent years, next-generation WAs, i.e. ones extracted from organic waste, have been used successfully^4^. The final result of washing, apart from a reduced total HM concentration, is an increase in the stability of the HMs remaining in the soil, resulting from the removal of mobile and potentially mobile forms of HMs^5,6^.

However, some WAs that efficiently remove HMs also change soil fertility and affect microbial activity, which plays an important role in soil organic turnover, element circulation, and phytotoxicity. The extent of these changes depends on the kind of WAs used. For example, Wang et al.^7^ reported that soil washing with weakly biodegradable EDTA removed macronutrients such as nitrogen, potassium, calcium or magnesium, causing the soil to lose its capacity to supply essential nutrients for plant growth. Other studies have also found that EDTA may adversely affect microorganisms and plants^8,9^. In a study by Guo et al.^10^, EDTA, FeCl_3_ and mixed chelators (EDTA, glutamic acid and citric acid) all decreased total and exchangeable nutrient content in washed soil (apart from P content). However, soil washed with mixed chelators retained more nutrients than that washed with FeCl_3_ or EDTA alone. Wang et al.^1^ found that biodegradable chelators (iminodisuccinic acid (ISA), glutamate-N,N-diacetic acid (GLDA), glucomonocarbonic acid (GCA) and polyaspartic acid (PASP)) removed Cd, Pb and Zn from farmland soil and mine soil with maximum removal efficiencies of 85.0, 55.0 and 64.0%, and 45.0, 53.0 and 32.0%, respectively. The capacity of ISA and GLDA to reduce the content of Cd, Pb, and Zn in the labile fraction was similar to that of EDTA. Although nutrient content (microbial biomass nitrogen and phosphorus) and enzyme activity (soil β-glucosidase, urease, acid phosphatase) had decreased in all treated soils, soil enzyme activities were 5.0–94.0% higher after treatment with biodegradable chelators than after treatment with EDTA.

However, the above results only deal with HM removal under batch soil washing, and research on column washing (as a simulation of soil flushing) is conducted less frequently. Moreover, the authors of column washing studies have tended to focus mainly on the efficiency of HM removal. For example, Yang et al.^11^ evaluated HCl and Na_2_EDTA for removal of Cd (12.8 mg/kg) and Pb (105.4 mg/kg) from contaminated soil. They showed that both WAs effectively removed Cd, but low concentrations of HCl did not effectively remove Pb, and at the optimal dose of Na_2_EDTA, only 45.4% of Pb was removed. In order to effectively remove both HMs, those authors tested sequential use of the optimal dose of HCl followed by the optimal dose of Na_2_EDTA. This sequential washing removed Cd and Pb with 87.3% and 73.2% efficiency, respectively. Other authors demonstrated good performance of biosurfactants for HMs removal from soil in column flushing. With the use of soap solution the removal efficiency of Cd, Cu, Pb and Zn was 62.0–69.0%, while with the use of rhamnolipids, it was 59.0–63.0%^12^.

Our previous studies have indicated that next-generation WAs in form of dissolved organics from sewage sludge remove HMs with high efficiency in both batch conditions and washing columns^4,6,13,14^. However, a holistic assessment of how these agents affect the functionality of remediated soil has only been done with soils washed under batch conditions^13^. Because soil flushing is more similar to washing in field conditions than batch washing, evaluation of the quality of flushed soil based not only on residual HM concentrations and the fate of residual HMs, but also on soil properties that enable recovery of its function should be performed before full-scale application of WAs. Until now, however, such a comprehensive assessment of soil after flushing has not been performed.

To address these shortcomings, the objective of this study was to comprehensively assess the quality of soil after column flushing. Additionally, we provide added value via a detailed comparison of the results of column flushing with previously published results of soil quality after washing under batch conditions^13^. The comparison involves the same soil and the same sludge-derived WAs (SS_WAs: dissolved organic matter, DOM; soluble humic-like substances, HLS and soluble humic substances, SHS). Moreover, the results obtained by soil washing and soil flushing with SS_WAs are compared to those obtained by washing with Na_2_EDTA, the most frequently used conventional non-biodegradable chelator. To the authors’ knowledge, this is the first study to comprehensively assess soil flushed with SS_WAs in a column experiment and the first paper to compare soil quality after remediation with soil flushing and soil washing.

## Materials and methods

### Soil

Sandy loam soil with low organic matter content was used in this study. Samples of surface soil (0–30 cm) were collected from an agricultural area (North-East Poland). After suitable laboratory preparation (air-drying, crushing, sieving), the soil (ca. 5 kg) was spiked with a mixture of Cu, Pb, and Zn (in form of nitrate salts) for concentrations typical in soil from a metallurgical area in Poland^15,16^. After spiking, the soil was incubated for 3 months at room temperature to reach equilibrium (earlier studies indicate that the most intense changes in the distribution of HMs in soil occur within 3 months from spiking^17–19^. Finally, the soil was air-dried and kept in a closed polyethylene container for further analyses and experiments. Details of the physico-chemical characteristics of the spiked soil are presented in Table 1.

**Table 1:** Physical and chemical properties of spiked soil (n = 3, ± standard deviation).

Physical properties			Chemical properties		
Sand	%	56.0 (± 1.6)	pH	–	6.4 (± 0.1)
Silt	%	39.0 (± 0.16)	OM	%	3.4 (± 0.08)
Clay	%	5.0 (± 0.21)	HS	mg/kg	10.9 (± 0.9)
Texture	–	Sandy loam	CEC	cmol ( +)/kg	17.2 (± 0.7)
Bulk density	g/cm^3^	1.2 (± 0.1)	Cu	mg/kg	7874.5 (± 23.1)
Porosity	–	0.5 (± 0.07)	Pb		1414.3 (± 11.6)
WHC	%	20.1 (± 1.6)	Zn		566.1 (± 4.2)

*OM* organic matter, *HS* humic substances, *CEC* cation exchange capacity, *WHC* water holding capacity.

### Washing agents

Soil flushing was performed with three SS_WAs: dissolved organic matter (DOM), soluble humic-like substances (HLS), soluble humic substances (SHS), as well as with Na_2_EDTA (Sigma-Aldrich, Germany). DOM, HLS, and SHS were prepared in a laboratory from municipal sewage sludge according to the procedure described in Kulikowska et al.^16^ and Klik et al.^6^.

The results presented here are part of a research project concerning soil remediation efficiency in batch conditions and in a column experiment using the same soil with the same HM concentrations. The results of the optimalization process and the batch washing results (SS_WA concentrations of 5 g TOC/L, Na_2_EDTA concentration of 0.005 M (0.6 g TOC/L), and a pH of 4.0 for all WAs) are presented in our previous study^13^. Under the extraction conditions of DOM, HLS and SHS from sewage sludge, the SS_WAs contained trace concentrations of Cu (1.1–4.7 mg/L) and Zn (0.7–3.6 mg/L). For comparison, concentrations of Cu and Zn in Na_2_EDTA solution were 0.7 and 1.6 mg/L, respectively. All WAs did not contain toxic Cd and Pb. On this basis, the WAs used for soil flushing do not cause secondary soil contamination with HMs.

### Soil flushing

Soil flushing was performed in a PVC column with an internal diameter of 30 mm and a length of 300 mm. The column was packed with 50 g of contaminated soil. To prevent disturbances in flow rate in the column, a double layer of clean filter material with different textures (gravel with ϕ 2–4 mm and sand with ϕ 1–2 mm) was placed at two heights of the column, i.e. in the inlet and outlet. At the bottom of the column, a membrane filter was inserted to protect the effluent from solids and turbidity. Detailed description of the column reactor was presented in Klik et al.^14^. Before the experiments, the column was saturated with deionized water for 1 h to remove entrapped air. Soil flushing was carried out at 2 different flow rates (0.5 mL/min and 1.0 mL/min), employing a peristaltic pump (Lead Fluid, BT600S). In the remaining parts of this paper, the following abbreviations are used when referring to the use of DOM, HLS, SHS and Na_2_EDTA as WAs at flow rates of 0.5 mL/min and 1.0 mL/min: DOM_0.5_, DOM_1.0_, HLS_0.5_, HLS_1.0_, SHS_0.5_, SHS_1.0_, Na_2_EDTA_0.5_, and Na_2_EDTA_1.0_.

The column eluate was collected regularly with an automatic sampling device after every hour during 24 h-flushing, which corresponded to 12.7–25.4 pore volumes.

### Characterization of soil before and after flushing

Soil quality was characterized on the basis of following indicators: pH, total Cu, Pb and Zn concentrations and individual HM mobility, concentration of soil organic matter, therein humic substances (HS) and their fractions (fulvic fraction, FF and humic acid, HA), ammonium nitrogen (NH_4_), available P, exchangeable K, Na, Ca, and Mg. Apart from above indicators, soil microbial activity (on the basis of dehydrogenase activity, DHA) and soil phytotoxicity (on the basis of *Triticum aestivum* seed germination and growth) were determined.

### Chemical indicators

The soil pH was measured in distilled water at 1:2.5 ratio using a pH-meter. Total contents of Cu, Pb, and Zn as well as macroelements (Na, Mg and Ca) were measured with a flame atomic absorption spectrometer (FAAS) (AA 280FS, Varian, Australia) after microwave mineralization of the soil in aqua reqia (HCl:HNO_3_, 3:1) (MarsXpress, CEM). TraceCERT®heavy metal standards for FAAS (Sigma-Aldrich, Saint Louis, MO, USA) were used to prepare the calibration curve. The accuracy of metal analysis by FAAS was validated by analyzing the reference material, CRM 142 R. The limits of detection (LOD) for individual Cu, Pb and Zn were 0.3, 1.6 and 0.3 mg/L, respectively. The limits of quantification (LOQ) of these HMs were 1.2, 4.8 and 0.9, respectively.

To assess soil quality in terms of its environmental risk, we focused on the analysis of HM concentration only in exchangeable and acid soluble fraction (F1) based on soil extraction with 0.11 M CH_3_COOH^20^. The F1 fraction includes weakly adsorbed HMs on soil constituents’ surface by electrostatic interactions, HMs that can be released by ion-exchange, and HMs co-precipitated with carbonates that can be remobilized after soil pH lowering. Based on the concentration of HMs in the F1 fraction and total HM concentration, the mobility factor (MF)^21,22^ was calculated. With the MF values, the level of potential HM mobility (very high mobility, high mobility, medium mobility, low mobility) was estimated. The complete HM distribution patterns in the flushed soil was presented in Klik et al.^6^.

The soil OM content was measured with Tiurin method^23^. HS, HA and FF were extracted from the soil with procedures cited in Kulikowska et al.^16^. Ammonium nitrogen, available P, and exchangeable K in soils were determined according to Polish Standards (PB 30ED.3 03.12.2012, PN-R-04023:1996, PN-R-04022:1996) as an outsourced analysis.

### Soil phytotoxicity and enzymatic activity

To establish soil phytotoxicity, wheat (*Triticum aestivum* L. cv. Herenda*)* seed germination and growth test was made^24^. The plant seeds were provided by a garden trading wholesale in Sieradz (Poland). The test was carried out in covered Petri dishes, in each of them 10 wheat seeds were placed. The dishes were kept in the dark at 20 ± 1 °C for 72 h. After the test, the plants were measured and only the seeds with a root tip of at least 1 mm in length were recognized as germinated. The phytotoxicity was expressed as an inhibition factor (*I*; in the latter part of manuscript, *I*_s_ means the shoot inhibition factor and *I*_r_ means the root inhibition factor)^1^:

1 $$I \left(\mathrm{\%}\right)=\frac{\left({L}_{c}-{L}_{t}\right)\times 100}{{L}_{c}},$$

where *L*_c_ is the average shoot/root length in the control sample (mm) and *L*_t_ is the average shoot/root length in the tested sample (mm). The soil unspiked with the HMs was used as a control sample.

Apart from *I*, germination rate (GR) was established^1,25^:

2 $$GR (\mathrm{\%})=\frac{{N}_{\mathrm{gs}} }{{N}_{\mathrm{ts}}}\times 100,$$

where *N*_gs_ is the number of germinated seeds and *N*_ts_ is the total number of seeds.

The analyses of soil phytotoxicity, including the collection of plant material, comply with the relevant institutional, national, and international guidelines and legislation.

The activity of soil microorganisms was assessed on the basis of soil dehydrogenase activity (DHA)^26,27^. Briefly, the soil was mixed with CaCO_3_, then shaken and incubated at 37 °C with 3% 2,3,5-triphenyltetrazolium chloride (TTC) and distilled water (20 h in the dark). Next, triphenyl formazan (TPF) was extracted from the sample with ethyl alcohol. The extract was filtered and the absorbance of TPF at 485 nm was measured (RayLeigh spectrophotometer). DHA was as expressed as μg TPF/g d.w.·h.

### Statistical analysis

The experiments on soil flushing were performed in duplicate, whereas chemical analyses of eluates and soil, in triplicate. Statistica 13.3 (Software Inc.) was used for processing the experimental data. Statistical comparisons between treatments were performed with one-way analysis of variance (ANOVA), followed by Tukey's honest significant difference (HSD) test.

## Results and discussion

### Residual HMs in the flushed soil and their mobility

One of the aims of soil remediation is a permanent and substantial reduction in the amount, toxicity or mobility of pollutants. In this study, many factors affected HM removal, such as the type of WA, the flow rate of the WA and the type of HM. In general, the residual HM contents in soil flushed at a flow rate of 1.0 ml/min. were significantly lower than those in soil flushed at 0.5 ml/min (p < 0.05).

**Figure 1:**
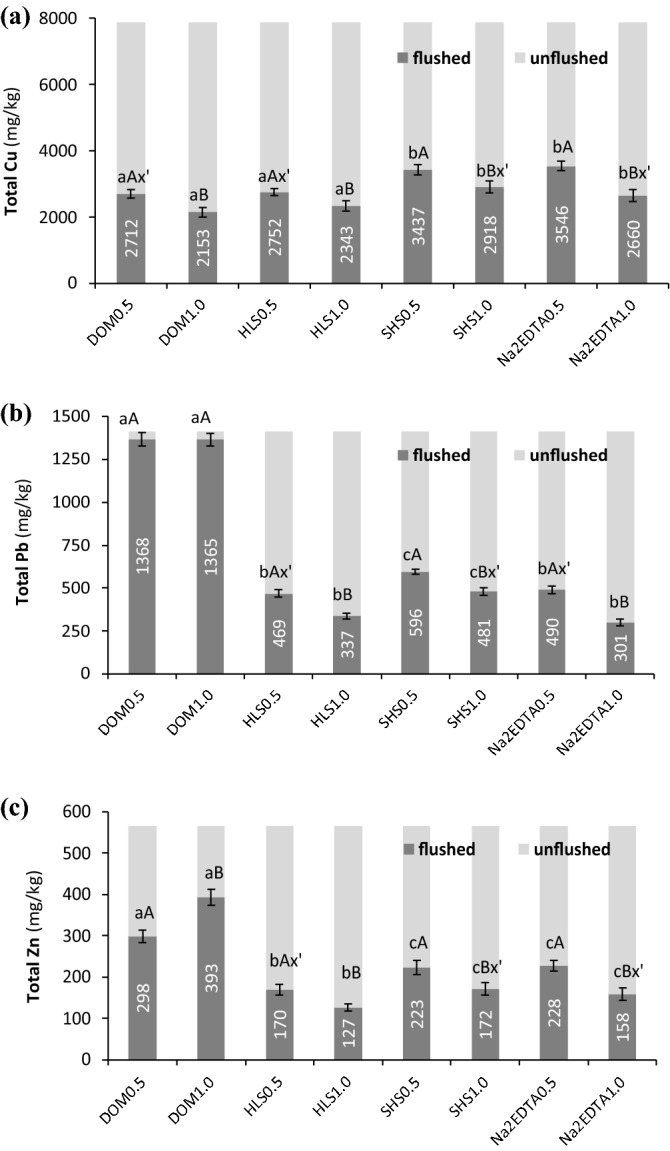
Total HM concentrations in soil before and after flushing with DOM, HLS, SHS and Na_2_EDTA: (**a**) Cu, (**b**) Pb, (**c**) Zn. Small letters (ab) indicate statistically significant differences in HM concentration between the WAs at one flow rate, capital letters (AB) indicate significant differences in HM concentration between the flow rates with one WA. The symbol x' means no statistically significant differences between treatments. The values in grey columns mean residual HM concentration in flushed soil.

With all WAs, the decrease in Cu content (2.2–3.7-fold) was larger than that of the other HMs (Fig. 1a). In soil flushed with DOM or HLS, the residual Cu content was lower than that in soil flushed with SHS or Na_2_EDTA. Although the amount of Cu removed from the soil was very high (5060.0 mg/kg, on average), its residual content exceeded the permissible level for soil according to national legislation (600.0 mg/kg)^28^.

Flushing with HLS, SHS and Na_2_EDTA decreased Pb content below the limit (600.0 mg/kg)^28^, but after flushing with DOM, the residual content of Pb in the soil exceeded the permissible value by 2.2-fold (Fig. 1b). This is because DOM extracted from sewage sludge contained low-molecular-weight organics and fulvic acids which have a stronger affinity for Cu than Pb^13,29^. HLS and Na_2_EDTA removed Pb most effectively, which means that HLS can be considered for remediating Pb-contaminated soil and can serve as a substitute for Na_2_EDTA, but DOM should not be used for remediating soil contaminated with this HM.

Zn is a coexisting HM in soil affected by the smelting industry in Poland, but its content is lower than that of Cu and Pb and below the permissible value (2000.0 mg/kg)^28^. In this study, all WAs decreased the total content of this HM. Soil flushed with DOM had a higher residual Zn content than soil flushed with other WAs (Fig. 1c).

It is not sufficient to assess soil quality only on the basis of total HM content, as the environment risk of a HM stems from its availability and mobility, not its total content. The content of HMs in the F1 fraction can be a good indicator of the quality of HM-contaminated soil and remediated soil, as HMs in this fraction have the highest mobility and availability and pose the greatest risk to the environment. In the contaminated soil in this study, the respective percent contents of HMs in the F1 fraction, termed the mobility factor (MF), were 86.0%, 74.0% and 76.0% for Cu, Pb and Zn, indicating very high environmental risk. Thus, the contents of the F1 fraction made the largest contribution to the overall removal of HMs from the soil.

**Figure 2:**
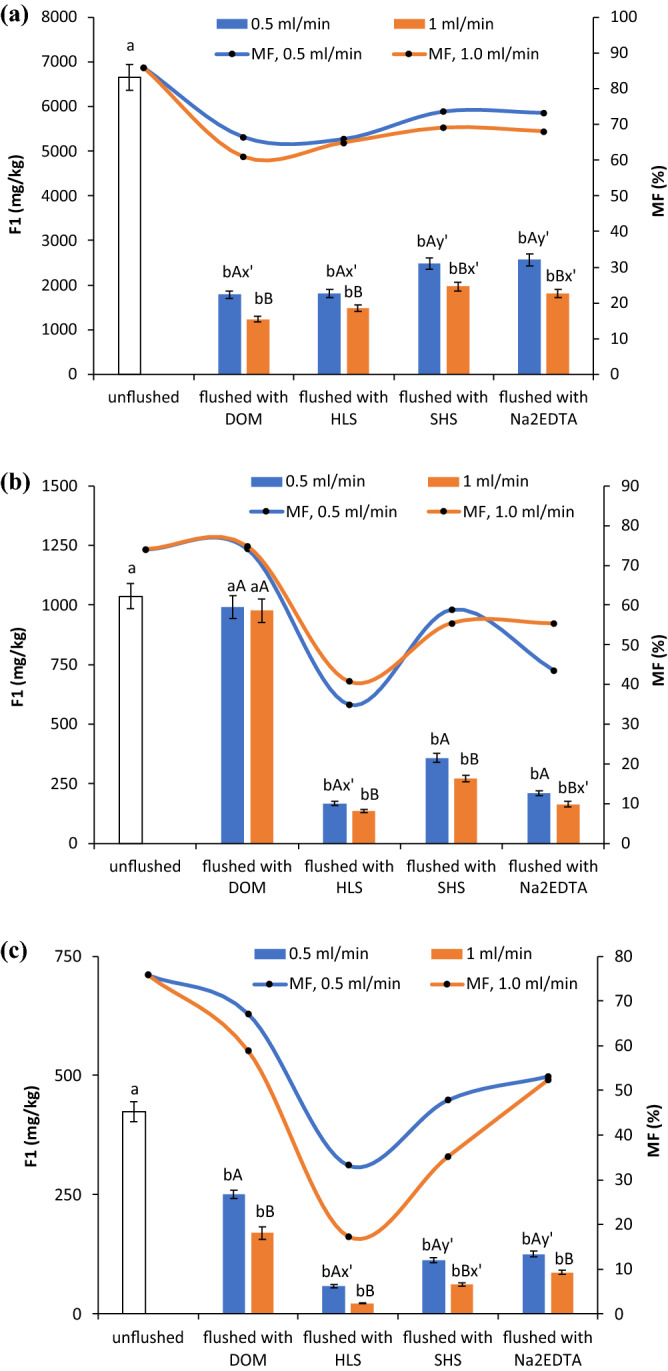
Changes in the content of the exchangeable and acid soluble fraction (F1) and the mobility factor (MF) of HMs in soil before and after flushing with DOM, HLS, SHS and Na_2_EDTA: (**a**) Cu, (**b**) Pb, (**c**) Zn. Small letters (ab) indicate statistically significant differences in the content of the F1 fraction between the unflushed and flushed soils, capital letters (AB) indicate significant differences in the content of the F1 fraction between the flow rates with one WA. The symbols x’ or y’ indicate no statistically significant differences between some treatments.

Although the total contents of HMs in contaminated soil differed considerably, the HMs posed similar risks to the environment, based on the MF values. Soil flushing markedly reduced the concentrations of HMs in the F1 fraction and the MF, except for Pb flushed with DOM (Fig. 2). After soil flushing at 1.0 ml/min, the MF was significantly lower than after soil flushing at 0.5 ml/min (p < 0.05). In soil washed with DOM at 1.0 ml/min, the Cu concentration in the F1 fraction (1236.0 mg/kg) was lower than in soil washed with other WAs, but its mobility was still very high (MF = 61.0%). HLS lowered Pb mobility more efficiently than the other WAs (MF = 35.0% at 0.5 ml/min, MF = 41.0% at 1.0 ml/min) (Fig. 2b). Particularly at the higher flow rate, HLS were also the most effective WA for decreasing Zn mobility from a very high (76.0%) to a medium level (MF = 17.0%) (Fig. 2c). Thus, for simultaneously removing all tested HMs and decreasing their mobility, HLS seem to be the most appropriate WA.

HMs were not completely removed under column flushing conditions. This could be a result of competition between individual HMs for active sites on the WAs, relatively quick saturation of the active sites with exchangeable HMs and sorption of the WAs in soil. Under batch conditions, in contrast, Borggaard et al.^30^ observed that EDTA and HS from processed cow slurry almost completely removed Cu from the exchangeable, carbonate-bound and oxide-bound fractions. Similarly, our previous study revealed that most of the mobile F1 fraction of Cu, Pb and Zn was removed with most of the tested SS_WAs and Na_2_EDTA under both batch and dynamic washing conditions. In the flushed soil, however, the respective MFs for Cu, Pb and Zn were 1.5–4.1-fold, 1.3–10.7-fold and 3.6–9.9-fold higher than in the washed soil^6^. Elimination of the most reactive HM fractions is desirable because these fractions are the most available to plants and the most easily leached.

### Changes in soil properties before and after flushing

As indicated by the analysis of soil characteristics (pH, OM, HS and their fractions, ammonium nitrogen and available micro- and macronutrients), soil flushing affected the properties of the soil, but the magnitude and direction of the change depended on the WA that was used.

The WAs that were used had acidic reactions, and as a result, the pH in the soil was lower after flushing (the pH decreased by 0.4, on average, after flushing with SS_WAs, and by 0.6 after flushing with Na_2_EDTA). With a particular WA (DOM, Na_2_EDTA), the pH after flushing at the tested flow rates did not differ significantly (p > 0.05) (Table 2).

**Table 2:** Comparison of pH, and selected macroelement concentrations (in mg/kg) in soil before and after flushing with DOM, HLS, SHS and Na_2_EDTA (standard deviation from the mean value, n = 3).

Characteristic	Unflushed	Flushed							
		DOM_0.5_	DOM_1.0_	HLS_0.5_	HLS_1.0_	SHS_0.5_	SHS_1.0_	Na_2_EDTA_0.5_	Na_2_EDTA_1.0_
pH	6.4 ± 0.1**a**	6.0 ± 0.1**bAx’**	5.9 ± 0.2**bAx’y’**	6.1 ± 0.2**aAx’**	5.9 ± 0.1**bAx’y’**	6.1 ± 0.2**aAx’y’**	6.0 ± 0.2**bAx’y’**	5.9 ± 0.1**bAx’y’**	5.8 ± 0.2**bAy’**
N-NH_4_	26.2 ± 3.1**a**	52.7 ± 4.1**bAx’**	56.6 ± 4.2**bA**	69.6 ± 4.7**bA**	57.9 ± 3.8**bBx’**	34.0 ± 3.0**aAy’**	29.7 ± 3.5**aA**	25.8 ± 3.1**aAx’**	27.0 ± 2.1**aAy’**
Available P	158.0 ± 11.9**a**	372.6 ± 20.7**bAx’**	391.1 ± 24.2**bAx’**	343.9 ± 16.7**bAx’**	364.2 ± 22.5**bAx’**	356.9 ± 19.6**bAx’**	382.6 ± 17.1**bAx’**	152.5 ± 22.2**aA**	160.1 ± 13.6**aA**
Exchangeable K	107.0 ± 12.6**a**	301.6 ± 12.1**bAx’**	316.3 ± 23.0**bAx’**	276.8 ± 14.8**bAx’**	290.5 ± 17.5**bAx’**	282.0 ± 11.0**bAx’**	294.8 ± 14.4**bAx’**	85.4 ± 13.7**aB**	79.3 ± 4.8**aB**
Exchangeable Na	72.0 ± 3.9**a**	233.8 ± 10.6**bAx’**	271.9 ± 19.5**bA**	529.8 ± 30.5**bA**	542.6 ± 30.5**bA**	664.9 ± 29.5**bA**	664.9 ± 29.4**bA**	192.8 ± 11.8**bAx’**	192.8 ± 10.5**bA**
Exchangeable Ca	849.0 ± 12.3**a**	520.5 ± 17.8**bAx’**	497.2 ± 24.8**bAx’**	524.1 ± 34.1**bAx’**	481.7 ± 27.2**bAx’**	544.5 ± 26.8**bA**	544.5 ± 24.7**bA**	480.1 ± 20.7**bAx’**	462.7 ± 18.5**bA**
Exchangeable Mg	90.0 ± 4.5**a**	78.5 ± 3.0**aAx’**	70.9 ± 3.5**bAx’**	77.6 ± 6.8**aAx’**	60.6 ± 3.2**bBx’**	116.9 ± 4.9**bA**	133.9 ± 9.5**bA**	68.2 ± 4.8**bAx’**	63.8 ± 5.2**bA**

For a given nutrient, different letters indicate significant differences in nutrient content in unflushed and flushed soil (ANOVA followed by Tukey’s HSD test, p < 0.05). Small letters indicate differences in nutrient content between unflushed and flushed soil, capital letters indicate differences in nutrient content in soil flushed at different flow rates with a given WA. The sign x' or y’ means no statistically significant differences between some treatments.

**Figure 3:**
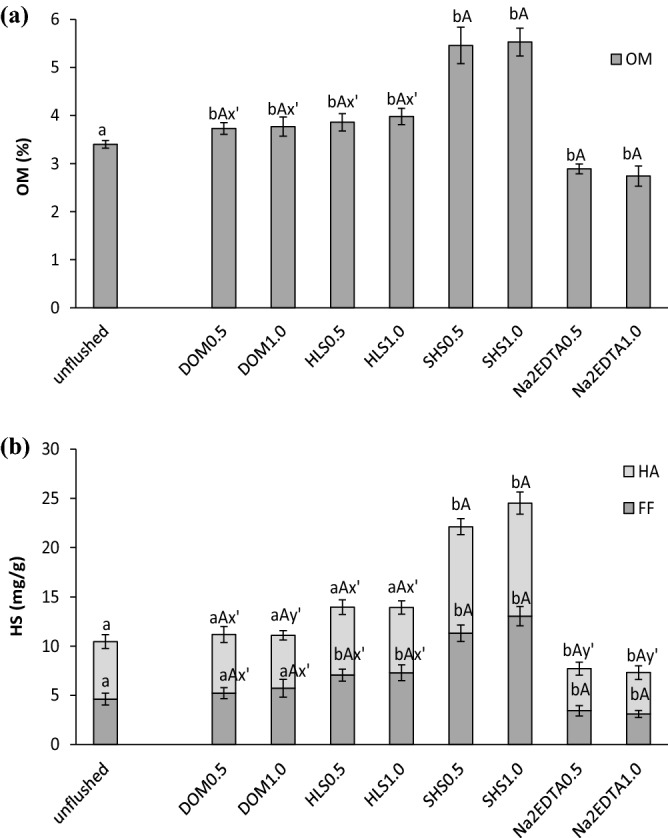
Changes in the content of organic matter (OM) (**a**), humic substances (HS) and their fractions, i.e. humic acids (HA) and fulvic fractions (FF) (**b**) in soil before and after flushing with DOM, HLS, SHS and Na_2_EDTA. Small letters (ab) indicate statistically significant differences in the content of OM, HA and FF between the unflushed and flushed soils, capital letters (AB) indicate significant differences between the flow rates with one WA. The symbols x' or y’ indicate no statistically significant differences between some treatments.

OM content was increased significantly (p < 0.05) by soil flushing with the SS_WAs, but it was decreased by flushing with Na_2_EDTA (Fig. 3a). In unflushed soil, OM content was 3.4%, and flushing with SHS increased OM content almost 1.6-fold (to 5.5%), whereas flushing with DOM and HLS increased it to a lesser extent (to 3.8%, on average). In contrast, flushing with Na_2_EDTA lowered OM content to 2.8%, on average. Flushing intensity (flow rate) did not appear to affect OM content. Other authors have also reported that soil flushing/washing with waste-derived WAs increases OM or organic carbon content. For example, Juwarkar et al.^31^ stated that organic carbon content in soil increased from 0.3% in contaminated soil to 0.4% after washing with a rhamnolipid biosurfactant produced by *Pseudomonas aeruginosa* strain BS2. However, reports indicate that soil washing with conventional WAs does not affect the OM content. For example, Gao et al.^32^ stated that in soil washed with FeCl_3_, citric acid and FeCl_3_ with citric acid, OM content did not change compared to unwashed soil. Zupanc et al.^33^ also reported unchanged soil OM content after soil washing with EDTA: the OM contents of the unwashed and remediated soil were 7.8 and 7.7%, respectively. Similar results were reported by Jelusic and Lestan^34^. Soil properties such as OM and CEC can influence the removal efficiency of HMs. An increase in OM in soil can enhance pollutant retention in soil^35^ and diminish their removal. An increase in OM in the flushed soil has a positive effect on soil fertility and soil health^36^.

This study investigated not only OM content in soil, but also that of humic substances (HS), humic acids (HA) and the fulvic fraction. Although it is known that HS positively affect the physicochemical properties of soil, an analysis of their content in washed/flushed soil is often omitted. It was found that soil flushing with HLS and SHS clearly increased soil HS content by 1.3-fold and over twofold, respectively (Fig. 3b). Flushing with DOM increased HS content only slightly. In contrast, flushing with Na_2_EDTA caused 0.7-fold decrease in HS content.

Whereas soil flushing with HLS and SHS increased soil HS content, it decreased the share of HA in HS. In unflushed soil, HA constituted 55.9% of HS and this decreased to 49.5% and 48.9% after flushing with HLS and SHS, respectively. However, it should be emphasized that despite the percent decreases, HA contents were higher in the flushed soils (6.8 mg/g, on average, after HLS and 11.1 mg/g, on average, after SHS) than in the unflushed soil (5.8 mg/g). Similar changes in FF content were noted, i.e., increases after flushing with HLS and SHS and a decrease after flushing with Na_2_EDTA. All these changes are consistent with what was observed in our previous study on soil washing with the same SS_WAs, but in batch conditions^13^.

HS content in soil is very important as these substances increase the quality and productivity of soil by improving its structure and ability to retain water and ensuring constant access to nutrients. HS affect the physical, chemical and biological properties of soils to a greater extent than other soil constituents. In the presence of Ca, an increase in HS content, especially that of HA, causes stable aggregates to form, which in turn, improves field water capacity, air capacity, soil porosity and permeability. Therefore, a loss of HS, especially HA, can increase soil compactness, decrease soil aeration, and lead to the presence of reductive chemical conditions^37^. In this context, it can be seen that soil flushing/soil washing with SS_WAs should not only remove HMs from soil, but also improve soil properties.

In soil, apart from OM and HS content, it is also important to consider the content of nutrients necessary for plant growth. The three principal macroelements are N, P and K, but Ca and Mg are also important. A lack of a clear decrease in ammonium content after soil remediation with Na_2_EDTA (Table 2) is a rather unusual phenomenon, as most studies have reported clear decreases in ammonium content. This is probably connected with the fact that most studies examined soil washing, not flushing. Our previous study that used the same soil also found a clear decrease in ammonium content after washing with Na_2_EDTA^13^.

Soil flushing with all SS_WAs increased available P, exchangeable K and exchangeable Na content in remediated soils and decreased exchangeable Ca and, in most cases, exchangeable Mg (Table 2). The use of Na_2_EDTA also decreased exchangeable Ca and exchangeable Mg content. Gao et al.^32^ also stated that soil washing with FeCl_3_, citric acid and FeCl_3_ with citric acid significantly decreased the content of exchangeable Mg. According to those authors, Ca^2+^ and Mg^2+^ compete with HMs for the binding sites of FeCl_3_ or citric acid during soil washing. As a result, Ca and Mg are removed along with the HMs. Wasay et al.^38^ made similar observations, but the amount of macronutrients that were washed out depended on the WA that was used. Those authors used salts of weak organic acids (citrate, tartrate, oxalate with citrate) and chelating agents (EDTA or DTPA) to remediate polluted soils in a column experiment. Four to five times more macronutrients, such as Ca, Mg and Fe, were leached with EDTA and DTPA than with citrate. Salts of tartrate also leached only a small amount of macro-nutrients. EDTA and DTPA form strong chelates with soil macronutrients that have much higher stability constants than those of chelates formed with citrate or tartrate. Therefore, EDTA and DTPA also extracted other soil nutrients, thereby decreasing the chemical value of the soil.

In our study, soil flushing with Na_2_EDTA did not noticeably change the content of ammonium nitrogen, available P or exchangeable K. Importantly, the flow rate of all the WAs did not appreciably affect the macronutrient content in the soil. These results indicate that the use of SS_WAs during column flushing can play an important role in preserving nutrients in soil.

### Dehydrogenase activity and plant growth in flushed soil

Soil enzyme activity reflects soil contamination but also can serve as an indicator of biogeochemical cycles, OM degradation, and soil remediation processes. Thus, soil enzyme activity can indicate soil quality and may be useful for indicating both the degree of deterioration of soil quality or the recovery of soil function after remediation. Dehydrogenase is a soil enzyme which is found in intact cells, but does not accumulate extracellularly in the soil. It is known that dehydrogenases participate in and assure the correct sequence of biochemical pathways in soil biogeochemical cycles, and thus provide information on the biological activity and microbial populations in soil. Based on the DHA, the quality of the soil and the degree of regeneration of degraded soils can be assessed. Moreover, DHA is sensitive to HMs presented in soil, including Cu, Zn and Pb. Therefore, in this study, DHA was chosen to analyze soil enzymatic activity after soil flushing, as was done in Klik et al.^13^ after soil washing in batch conditions.

**Figure 4:**
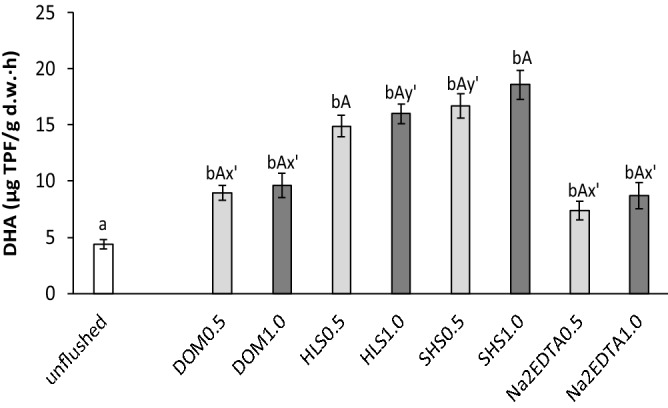
Changes in dehydrogenase activity (DHA) in soil before and after flushing with DOM, HLS, SHS and Na_2_EDTA. Small letters (ab) indicate statistically significant differences in DHA between the unflushed and flushed soils, capital letters (AB) indicate significant differences between the flow rates with one WA. The symbols x' or y’ indicate no statistically significant differences between tested soils.

In the contaminated soil, DHA was very low, 4.4 µg TPF/g d.w.·h. In soil flushed with Na_2_EDTA at 0.5 ml/min and 1.0 ml/min, DHA increased by only ca. 1.7–2.0-fold, to 7.4 µg TPF/g d.w.·h and 8.7 µg TPF/g d.w.·h, respectively. After flushing with SS_WAs, particularly with HLS and SHS, DHA was markedly higher (14.9–16.0 µg TPF/g d.w.·h and 16.7–18.6 µg TPF/g d.w.·h, respectively) (Fig. 4). Lower DHA after flushing with DOM resulted mainly from low efficiency of this WAs in Pb removal, mainly exchangeable and acid soluble fraction (F1), as this fraction have the highest mobility and availability and pose the greatest risk to the environment.

Although, in this study, DHA increased seriously after soil flushing, our previous study with the same soil and WAs, showed that soil washing enabled to obtain higher degree of soil regeneration: in soil after DOM, SHLS and SHS equalled 21.1 µg TPF/g d.w.·h, 30.7 µg TPF/g d.w.·h and 34.8 µg TPF/g d.w.·h, respectively^13^ and were about twofold higher than after soil flushing (this study). The smaller increase in DHA after washing with Na_2_EDTA may be caused by the chelating agent adsorbing on the soil surface^39^ and negatively affecting soil enzyme activity.

The increase in DHA after soil washing/flushing with HLS and SHS may have been due to the decrease in HM content in remediated soil, especially in the F1 fraction, and to the improvement in soil fertility, especially the increase in soil OM. An increase in OM content can increase DHA by increasing the amount of substrate for the enzymes. Although the contents of soil OM were similar after flushing (the present study) and washing^13^, DHA was lower after flushing. This is probably because the contents of HMs after soil remediation were higher in the present study than in Klik et al.^13^.

### Soil phytotoxicity

To evaluate the usability of soil remediated by column flushing with SS_WAs and Na_2_EDTA, tests involving seed germination (germination rate, GR) and growth of wheat (*Triticum aestivum*) roots and shoots were used. These tests are commonly used for assessing toxicity in environmental samples^1,7,40^.

In unflushed soil, the GR was 0, meaning that none of the seeds germinated, due to the very high total Cu and Pb concentrations which inhibited seed germination. After flushing with all the tested WAs, the seeds germinated. However, the percentage of germinated seeds was higher in soil flushed with SS_WAs (GR 76.7–86.7%) than in soil flushed with Na_2_EDTA (GR 63.3–66.7%) (Table 3). Other authors have reported that, although soil polluted with Cd, Pb and Zn inhibits seed germination to some extent (GR on the level ca. 40.0–70.0%), it does not inhibit germination completely^1^. They found that, after washing with GLDA and ISA, the GR increased by 13–40%. These increases are smaller than those in the present study because, in their study, seed germination was not completely inhibited.

**Table 3:** Comparison of germination parameters in soil before and after flushing with DOM, HLS, SHS and Na_2_EDTA.

Soil	GR (%)
Control sample	100.0
Unflushed	0.0
**Flushed**	
DOM_0.5_	76.7
DOM_1.0_	80.0
HLS_0.5_	83.3
HLS_1.0_	86.7
SHS_0.5_	76.7
SHS_1.0_	80.0
Na_2_EDTA_0.5_	63.3
Na_2_EDTA_1.0_	66.7

It is worth noting that, in the study presented here, even though the Cu and Pb concentrations in the F1 fraction were lower after flushing with Na_2_EDTA than after flushing with SHS, the GR was lower after Na_2_EDTA flushing. This may indicate that chelating agents not only decrease soil enzyme activity but also inhibit seed germination. Inhibition of seed germination by residual chelating agents may be caused by a lack of the substances and energy needed for seed germination, as indicated by reduced breakdown of starch and proteins in seed storages in soil containing residual EDTA, which has limited biodegradability^1,8,41^.

**Figure 5:**
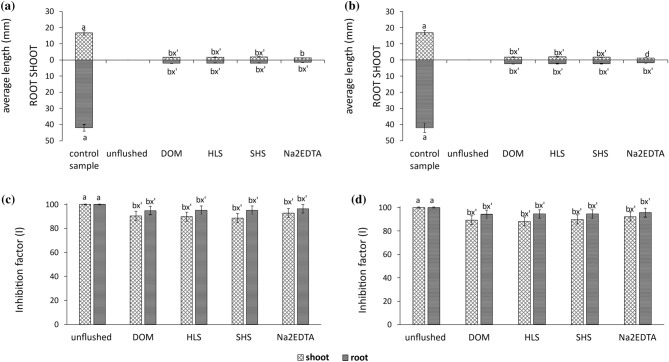
Changes in the lengths of wheat shoots and roots with inhibition factor (I) in soil before and after flushing with DOM, HLS, SHS and Na_2_EDTA: 0.5 ml/min (**a**,**c**), 1.0 ml/min (**b**,**d**). As a control sample, the unspiked soil was used. Small letters (ab) indicate statistically significant differences between the unflushed and flushed soils, the symbol x' or y’ indicates no significant differences between tested soils.

In the present study, the GR in unflushed soil was 0%. Soil flushing substantially improved the GR, even though the GR values were still lower in the flushed soils (63.0–67.0% after Na_2_EDTA; 83.0–87.0% after HLS) than the value in the control (unspiked) sample (100%). There was less improvement in shoot and root lengths after flushing (Fig. 5a,b), and the corresponding inhibition factors remained high (shoot inhibition, *I*_s_, and root inhibition, *I*_r_) with values in range of 88.0–96.0% (Fig. 5c,d). Although some authors have indicated that roots are more sensitive than shoots to changes in soil after washing^42^, in the present study, both appeared to be very sensitive to the effects of the HMs remaining in the flushed soil, as flushing did not substantially stimulate their growth. Similarly, Feng et al.^25^ reported that the length of rice roots in soil washed with two plant extracts (from *Fagopyrum esculentum* and *Fordiophyton faber*) and EDTA did not differ in a statistically significant manner from that of rice roots in contaminated soil.

### Comparison of soil quality: soil flushing vs. soil washing

Table 4 presents selected properties in the contaminated soil and the flushed soil in the present study, as well as these properties in soil after batch washing with DOM, HLS, SHS and Na_2_EDTA^13^. When using SS_WAs, the decrease in pH was smaller with soil flushing than with soil washing, but the increase in OM content in soil, and in the content of the HS, FF and HA fractions was larger with soil flushing. When applying Na_2_EDTA, more soil OM was lost during flushing than during washing.

**Table 4:** Comparison of the quality for contaminated, flushed and washed soil with DOM, HLS, SHS and Na_2_EDTA.

Soil characteristic	Contaminated soil	Flushed soil				Washed soil*			
		DOM_1.0_	HLS_1.0_	SHS_1.0_	Na_2_EDTA_1.0_	DOM	HLS	SHS	Na_2_EDTA
pH	6.4	− 0.5	− 0.5	− 0.4	− 0.6	− 0.8	− 0.7	− 0.9	− 1.2
Organic matter (%)	3.4	+ 0.4	+ 0.6	+ 2.1	− 0.7	+ 0.2	+ 0.4	+ 1.7	− 0.4
HS (mg/g)	10.7	+ 0.5	+ 3.9	+ 11.4	− 2.9	+ 0.2	+ 2.4	+ 9.2	− 1.9
FF (mg/g)	4.6	+ 1.1	+ 2.7	+ 8.4	− 1.5	+ 0.4	+ 1.8	+ 5.1	− 0.7
HA (mg/g)	5.8	− 0.5	+ 0.8	+ 5.6	− 1.6	+ 0.1	+ 0.8	+ 4.4	− 1.0
NH_4_ (mg/kg)	26.1	+ 30.4	+ 31.7	+ 3.5	− 0.8	+ 44.4	+ 65.7	+ 11.3	− 1.9
Available P (mg/kg)	158.0	+ 233.1	+ 206.2	+ 224.6	+ 2.1	+ 268.0	+ 249.0	+ 241.0	+ 5.0
Exchangeable K (mg/kg)	107.0	+ 209.3	+ 183.5	+ 187.8	− 27.7	+ 288.0	+ 241.0	+ 274.0	− 45.0
Exchangeable Mg (mg/kg)	90.0	− 19.1	− 29.4	+ 43.9	− 26.2	− 13.0	− 12.0	+ 215.0	− 11.0
Exchangeable Ca (mg/kg)	849.0	− 351.8	− 367.3	− 304.5	− 386.3	− 453.0	− 470.0	− 7.5	− 431.0
Exchangeable Na (mg/kg)	72.0	+ 199.9	+ 470.6	+ 592.9	+ 120.8	+ 267.0	+ 501.0	+ 666.0	+ 98.0
Total Cu (mg/kg)	7739.2	− 5709.1	− 5449.1	− 4891.2	− 5082.2	− 7205.9	− 6881.3	− 6162.3	7086.2
Total Pb (mg/kg)	1401.1	− 76.3	− 1072.0	− 909.4	− 1105.2	− 394.3	− 1132.0	− 889.4	1326.6
Total Zn (mg/kg)	559.4	− 185.1	− 431.3	− 383.0	− 393.1	− 485.4	− 504.8	− 499.1	− 526.6
Cu in F1 fraction (mg/kg)	6655.2	− 5418.9	− 5168.9	− 4687.2	− 4847.0	− 6520.5	− 6268.9	− 5607.2	− 6547.0
Pb in F1 fraction (mg/kg)	1036.6	− 45.5	− 902.5	− 764.0	− 872.8	− 305.5	− 952.5	− 746.0	− 1032.8
Zn in F1 fraction (mg/kg)	423.7	− 172.5	− 401.6	− 361.8	− 336.9	− 418.7	− 421.0	− 421.7	− 421.5
DHA (µg TPH/g d.w.·h)	4.4	+ 5.2	+ 11.6	+ 14.2	+ 4.3	+ 16.7	+ 26.3	+ 30.4	+ 4.0
GR (%)	0.0	+ 80.0	+ 86.7	+ 80.0	+ 66.7	+ 100.0	+ 100.0	+ 100.0	+ 100.0
I_s_ (%)	100.0	− 10.7	− 11.9	− 10.1	− 7.7	− 129.4	− 140.0	− 140.6	− 99.4
I_r_ (%)	100.0	− 5.7	− 5.5	− 5.5	− 4.3	− 96.7	− 111.6	− 105.6	− 68.0

The sign ‘−‘ and ‘ + ’ means a decrease or an increase, respectively in the value of a given soil characteristic in comparison to contaminated soil.

*Klik et al.^13^.

Overall, the fertility of flushed soil was lower than that of washed soil (with the exception of OM and HS content). For example, although using SS_WAs with both methods increased soil content of NH_4,_ available P, exchangeable K and exchangeable Na, the increases were smaller with soil flushing. However, the loss of exchangeable Ca was smaller with soil flushing than with soil washing, except when SHS was used. Except for HLS, washing or flushing with all WAs decreased the content of exchangeable Mg. With regard to HLS, soil washing increased exchangeable Mg content to a greater extent than soil flushing.

Although soil flushing decreased total HM content in the soil, soil washing was more effective. The reasons for the lower HM removal in the column flushing may be a shorter time for the HM in soil to react with WA in comparison to batch washing^43^. With most of the tested HMs and WAs, the HM concentration in the F1 fraction was higher after soil flushing than after soil washing. However, when using SHS, similar amounts of Pb were removed from the F1 fraction with both techniques; likewise, when using HLS, similar amounts of Zn were removed from this fraction with these techniques. DOM should not be recommended for Pb removal via soil flushing or soil washing because of the small amount of this HM that it removed from the F1 fraction and in total.

Both soil flushing and soil washing considerably improved the germination rate of *T. aestivum*. When flushing the soil, this effect was more pronounced with the SS_WAs than with Na_2_EDTA. Due to its greater content of residual HMs and higher HM mobility, soil flushed with the tested WAs was more phytotoxic (based on the *I*_s_ and *I*_r_ factors) than soil washed with these WAs.

The quality of flushed soil was better than that of washed soil with regard to pH and the content of OM, including HS and their fractions. Despite the considerable decrease in HM content in the flushed soil, the residual HM content in the F1 fraction was relatively high. Thus, soil flushed with these WAs would require more monitoring for potential environmental risk than washed soil.

## Conclusions

The advantage of using SS_WAs over Na_2_EDTA under flushing conditions is that they remove HMs and decrease HM mobility with better or similar efficiency. For Cu removal, flushing with DOM was most effective. However, DOM should not be used to remediate Pb-contaminated soil, due to its very low effectiveness, whereas HLS and Na_2_EDTA removed this HM most effectively. Independently of the WA that was used, the total residual content of HMs and the contents of individual HMs in the mobile fraction were significantly lower after treatment at the higher flushing rate than after treatment at the lower rate.

The use of SS_WAs during column flushing can help to preserve nutrients in soil. Soils flushed with DOM, HLS and SHS were enriched in organic matter content, including that of humic substances, content of nitrogen and phosphorus, which are very often the limiting nutrients in soils, as well as in magnesium content. Moreover, the soil flushed with SS_WAs showed better germination capacity and microbial activity that soil flushed with Na_2_EDTA. However, flushing with SS_WAs only improved shoot and root lengths by a small amount, and the corresponding inhibition factors remained high. Flow rate did not appear to affect DHA activity, soil toxicity indicators or the fertilizing properties of the soil.

Although soil washing with SS_WAs removes HMs from soil more efficiently than soil flushing with these WAs, soil flushing has more desirable effects on the pH, organic matter content, and the contents of HS, FF and HA than soil washing.
